# Enhanced efficiency of crystalline Si solar cells based on kerfless-thin wafers with nanohole arrays

**DOI:** 10.1038/s41598-018-21381-2

**Published:** 2018-02-22

**Authors:** Hyeon-Seung Lee, Jaekwon Suk, Hyeyeon Kim, Joonkon Kim, Jonghan Song, Doo Seok Jeong, Jong-Keuk Park, Won Mok Kim, Doh-Kwon Lee, Kyoung Jin Choi, Byeong-Kwon Ju, Taek Sung Lee, Inho Kim

**Affiliations:** 1 Center for Electronic Materials, Korea Institute of Science and Technology, Seoul, 02792 Republic of Korea; 2 School of Electrical Engineering, Korea University, Seoul, 02841 Republic of Korea; 3 Advanced Analysis Center, Korea Institute of Science and Technology, Seoul, 02792 Republic of Korea; 4 Photo-electronic Hybrids Research Center, Korea Institute of Science and Technology, Seoul, 02792 Republic of Korea; 5 School of Materials Science and Engineering, Ulsan National Institute of Science and Technology (UNIST), Ulsan, 44919 South Korea

## Abstract

Several techniques have been proposed for kerfless wafering of thin Si wafers, which is one of the most essential techniques for reducing Si material loss in conventional wafering methods to lower cell cost. Proton induced exfoliation is one of promising kerfless techniques due to the simplicity of the process of implantation and cleaving. However, for application to high efficiency solar cells, it is necessary to cope with some problems such as implantation damage removal and texturing of (111) oriented wafers. This study analyzes the end-of-range defects at both kerfless and donor wafers and ion cutting sites. Thermal treatment and isotropic etching processes allow nearly complete removal of implantation damages in the cleaved-thin wafers. Combining laser interference lithography and a reactive ion etch process, a facile nanoscale texturing process for the kerfless thin wafers of a (111) crystal orientation has been developed. We demonstrate that the introduction of nanohole array textures with an optimal design and complete damage removal lead to an improved efficiency of 15.2% based on the kerfless wafer of a 48 μm thickness using the standard architecture of the Al back surface field.

## Introduction

Si wafers for crystalline Si solar cells, produced by multi-wire sawing the Si ingot grown by a Czochralski method, have been consistently thinner to lower cell cost by reducing material consumption^1–3^. The thickness of Si wafers can be reduced by using multi-wire saws of smaller diameters^4^. However, it is known that a minimum thickness of Si wafers manufactured by a multi-wire sawing method is limited to approximately 80 μm. This is because a wafering yield is greatly reduced and the Si wastes are considerable when a Si wafer becomes thinner. In this regard, there has been numerous efforts to fabricate thin Si wafers below 50 μm with negligible wafering material losses, and this technique is termed kerfless wafering^5^.

Several kerfless wafering techniques have been proposed: epitaxial Si lift-off, stress-induced spalling, and smart-cut. Using an epitaxial Si lift-off technique, a thin wafer is epitaxially grown on a porous seed Si wafer by atmospheric chemical deposition (APCVD) and exfoliated from the parent seed wafer^6–10^. Epitaxial growth of high quality Si wafers, comparable to high performance CZ wafers, was demonstrated, and high efficiency of 21.2% based on a 35 μm thickness Si wafer was achieved^11^. However, this technique has high process complexity due to the production of porous Si and requires faster Si growth rates for commercialization. Many research works are underway in order to tackle these issues^12^. The stress-induced technique or SLIM-cut employs a stress induced layer on the Si wafer, and the stress is activated by thermal expansion mismatch between the stress layer and the Si wafer for spalling of thin wafers^13^. Recently, a novel method of electrodeposit-assisted stripping (EAS) has been developed to minimize the formation of micro-structural defects during the SLIM-cut process^14^. In the EAS process, a thin stress layer is electro-deposited at room temperature, and the lattice mismatch between the stress layer and the wafer induces a large stress field, which causes the lift-off of a thin Si wafer without high temperature annealing.

Another kerfless wafering technique based on a smart cut technique invented in 1990’s was attempted to fabricate kerfless wafers of tens of micrometer thickness for Si solar cells using a MeV proton implanter^15,16^. In this technique, a proton beam with a MeV energy is implanted into donor wafers^17^. The implanted protons are penetrated into a certain depth of the donor wafers depending on the proton acceleration energy. The implanted wafers are subsequently annealed to be exfoliated by hydrogen micro-bubble formation and crack propagation^18,19^. The proton induced exfoliation technique is a relatively simple and clean vacuum process compared with the epitaxial Si lift-off method^20^. In this technique, a critical proton dose for exfoliation of thin wafers relies on the crystal orientation of the parent Si wafers. The (111) Si surface has the lowest surface energy per unit area and in turn, the lowest fracture toughness; thus, the lowest critical proton dose is required for the kerfless wafering of the (111) Si wafers. Because the critical ion dose is directly related with manufacturing throughput time, the wafering of the (111) crystal orientation is most economically feasible. This limitation in the choice of crystal orientation pose challenges in light trapping and surface passivation. Also, the ion implanter of MV acceleration voltage and mA ion current is required for fabrication of the ultra-thin wafers with a thickness of 20 to 50 μm. The relatively high cost of special proton implanter compared with other wafering methods is one of the barriers for the commercialization of the proton induced exfoliation technique.

The implanted protons collide with Si host atoms while penetrating the donor wafer, losing the acceleration energy, resulting in creation of structural defects^21^. Most of the defects generated near the implantation surface of the wafer are easily removed by thermal annealing^20^. However, secondary defects such as dislocation loops and platelets formed at the ends of projected ranges are hardly removed^20–23^. For this reason, such defects are removed by etching a specific thickness of the cleaved wafers. Because the formation of the secondary defects leads to Si material losses, a thickness of the secondary defect zone induced implantation needs or end-of-range (EOR) defect zone to be analyzed^21^. However, a rare study on the EOR defect zone thickness induced by the MeV accelerated proton beams has been reported. Furthermore, the reported efficiency of Si solar cells based on kerfless thin wafers exfoliated by proton implantation is 13.2%^20^. The reported cell was based on a kerfless wafer of a 40 μm thickness and (111) crystal orientation with no texturing^20^. A critical proton dose for proton induced exfoliation relies on a crystal orientation of Si wafers. The (111) crystal orientation is preferred for kerfless wafering because a critical proton dose for a (111) wafer is much smaller than the proton dose for a (100) wafer. Conventional micro pyramid texturing is not applicable to a (111) crystal orientation wafer^24,25^. One of the main reasons for low-efficiency solar cells based on kerfless wafers is the difficulty in texturing of (111) oriented wafers^26,27^.

In this study, we analyze an EOR defect zone thickness induced by proton implantation and develop a texturing process for the kerfless-thin wafers of a (111) crystal orientation. We demonstrate proton induced damages in exfoliated wafers are nearly completely removed by thermal annealing and subsequent chemical etch. An EOR defect zone was analyzed with different techniques by SEM and TEM observations and minority carrier lifetime (MCLT) measurements. With a complete removal of implantation damages, we achieved an efficiency of 14.1% based on the kerfless thin wafers of a 48 µm thickness. We also show that introduction of nanohole texturing fabricated by laser interference lithography has resulted in an improved efficiency of 15.2%. Lastly, we discuss the effect of rear side modifications for effective light trapping in thin kerfless wafers.

## Results and Discussion

### Proton implantation exfoliation

**Figure 1:**
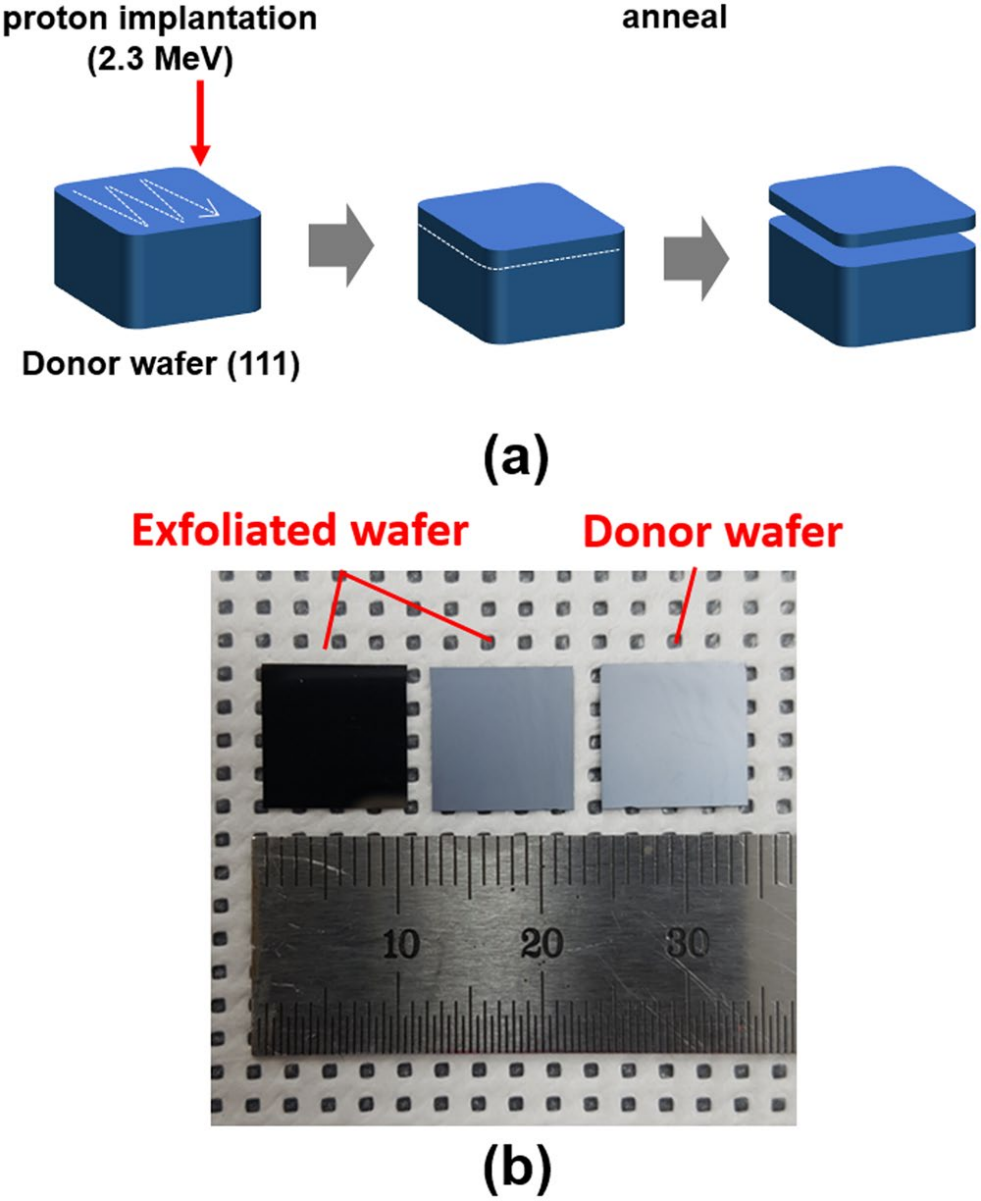
(**a**) Illustrations of the proton induced exfoliation process steps for kerfless wafering. (**b**) The photograph images of exfoliated wafers and a donor wafer. The left wafer is an exfolidated one with the cleaved surface facing down. The wafer in the center is an exfoliated one with the cleaved surface facing up. The righ wafer is an donor wafer with the cleaved sufrace facing up.

We fabricated kerfless wafers by using a proton induced exfoliation (PIE) technique. The Si wafers of a (111) crystal orientation were implanted with accelerated protons, and the donor wafers were subsequently annealed to produce the ultrathin wafers of a 58 μm thickness as illustrated in Fig. 1(a). The photograph images of the cleaved and donor wafers are shown in Fig. 1(b). Implanted protons make collisions with the host atoms of the Si donor wafers and induce multiple defects such as point, line and plane defects^21,28,29^. High temperature annealing above 700 °C removes most of point defects^20^; however, the planar defects of the shape of platelets which are formed during implantation and annealing are not removed by thermal treatment. The distribution and density of such defects play a main role in an exfoliation process because ion cutting is prone to occur in a heavily damaged region where the density of the platelets is the highest in the donor wafers. The distributions of the platelets are determined by multiple parameters such as the dose, acceleration voltage of implanted protons, and temperature of an irradiation stage^30^.

**Figure 2:**
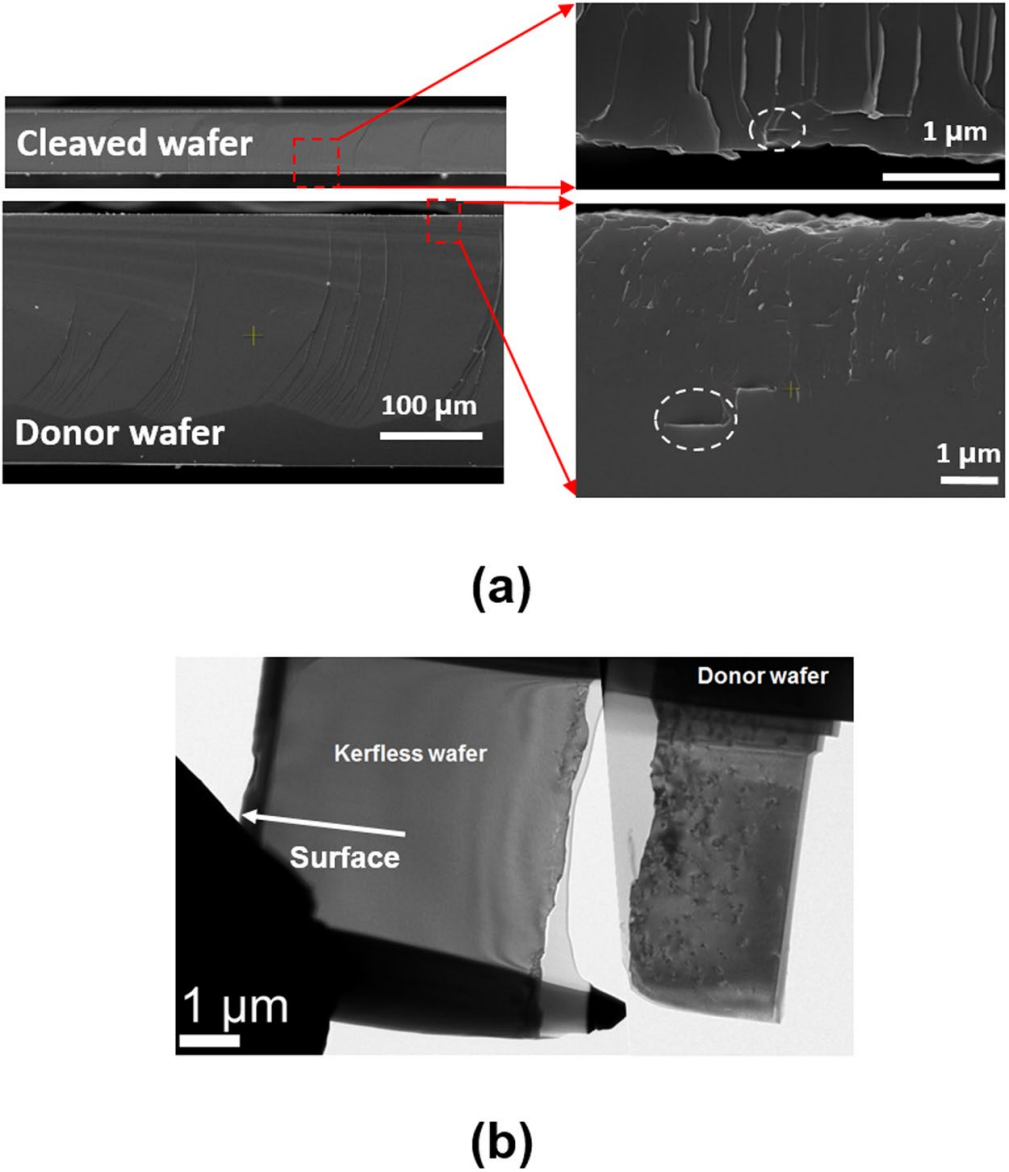
(**a**) Cross-sectional SEM images of the cleaved and donor wafers. The celaved sides on both of the wafers are magnified. One of the platelet defects are denoted by dashed circles in both of the cleaved and donor wafers. (**b**) Corss-sectional TEM images of the kerfless and donor wafers. The white arrow denotes the kerfless wafer surface side.

We observed the cross section of cleaved and donor wafers by SEM and TEM as shown in Fig. 2. The thickness of the cleaved wafer is 58 μm, which is confirmed by the SEM image. The platelets sizes ranging from hundreds nanometer to a micrometer are observed at both of the cleaved and donor wafers. The orientation of most platelets are parallel to the surface of the donor wafer, indicating that their crystal orientation is (111). In the case of the cleaved wafer, most platelets are observed within a depth of 1 μm from the cleaved surface while the platelets in the donor wafer are observed even as deep as 5 μm, indicating the ion cutting does not occur in the middle of the implantation damage zone.

A TEM analysis provide more detailed view about defect distributions as shown in Fig. 2(b). The cross sectional TEM images show that the implantation damage zone is composed of two regions: (i) an upper heavily damaged zone, (ii) a lower lightly damaged zone. The TEM analysis reveals that the ion cut occurs in the upper heavily damaged region. In good agreement with the SEM results, the donor wafer has a greater number of the defects in the region as deep as 3 μm, whereas the kerfless wafer has the defects only near the cleaved surface. This result indicates that the damaged regions at both sides of the kerfless wafer should be removed asymmetrically. The ion cutting is reported to occur due to agglomeration and propagation of platelets^31^. Distributions of implantation induced defects and hydrogen do not coincide and in general, the peak of hydrogen concentrations lies deeper than that of the defects. It is reported that the out-of-tensile strain induced by defects and implanted hydrogen plays a main role in the ion cut location^30^. When hydrogen dose is relatively low, the implantation induced damages more contribute to the out-of-tensile strain. This leads to the ion cutting in the upper damaged region.

**Figure 3:**
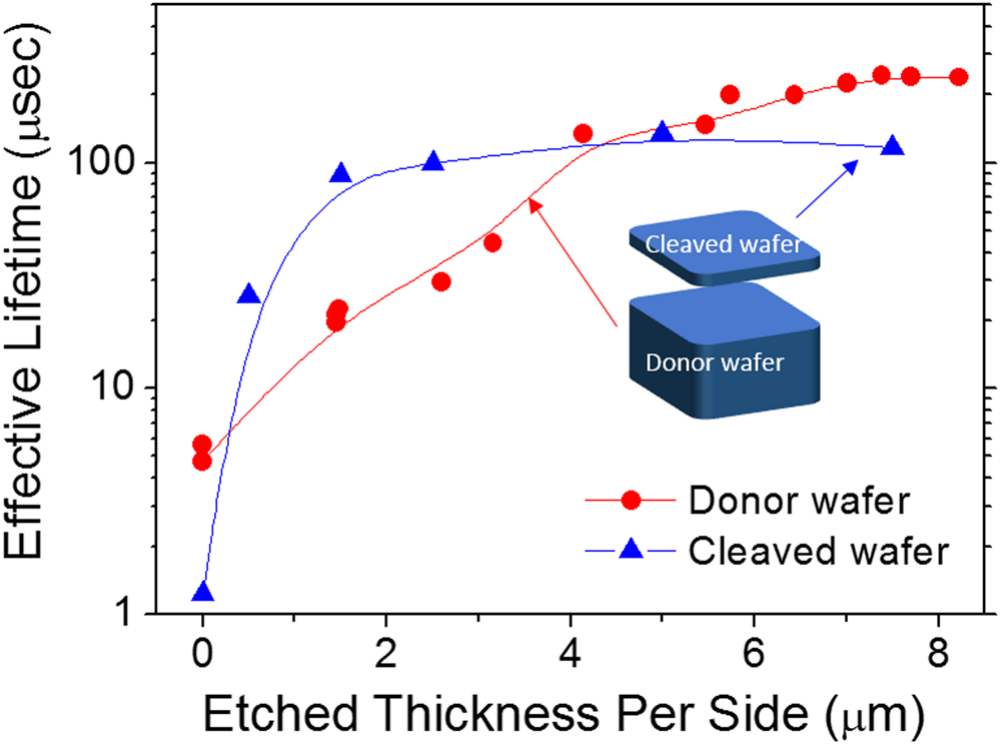
Effective minority carrier lifetimes of cleaved and donor wafers with wafer thinning by chemical etching of both sides of the wafers.

The complete damage removal of the kerfless wafer is essential for high efficiency solar cell fabrication because such damage induced defects serve as strong carrier recombination centers. The damage removal process with HNA (HF:HNO_3_:CH_3_COOH) solution was performed by varying etching time, and the measured effective lifetime of the cleaved and donor wafers is shown in Fig. 3. The as-cleaved wafers and donor wafers show low effective lifetimes shorter than 10 μsec, but the effective lifetime increases rapidly with increasing the etched thickness. As a reference, an effective lifetime of a bare wafer with no implantation was measured. In order to take a thickness effect into consideration, the bare wafer was chemically thinned to a 48 μm thickness with KOH, and its effective lifetime was measured. The bare wafer of a 300 μm thickness shows an effective lifetime of ~300 μsec while the chemically thinned wafer of a 48 μm thickness exhibits a lower value of ~130 μsec.

The thickness of an EOR defect zone can be defined as the minimum removal thickness at which the implanted wafers reach 80% of the effective lifetimes of the reference wafers of the same thickness. The thickness of an EOR defect zone by our definition is 2.5 μm and 7.4 μm for the cleaved and donor wafers, respectively. Thus, the total thickness of the EOR defect zone induced by implantation is approximately 10 μm out of a 58 μm of an initial cleaved wafer thickness; thus, nearly 17% of the cleaved wafer thickness needs to be removed for recovery of the effective lifetime of the cleaved wafers. The recyclability of the donor wafer is another key to the cost savings in the kerfless wafering technology. We have successfully exfoliated the kerfless-thin wafers from the used donor wafers, and the detailed results are described in supplementary information.

### Cell fabrication and performances based on kerfless thin wafers

**Figure 4:**
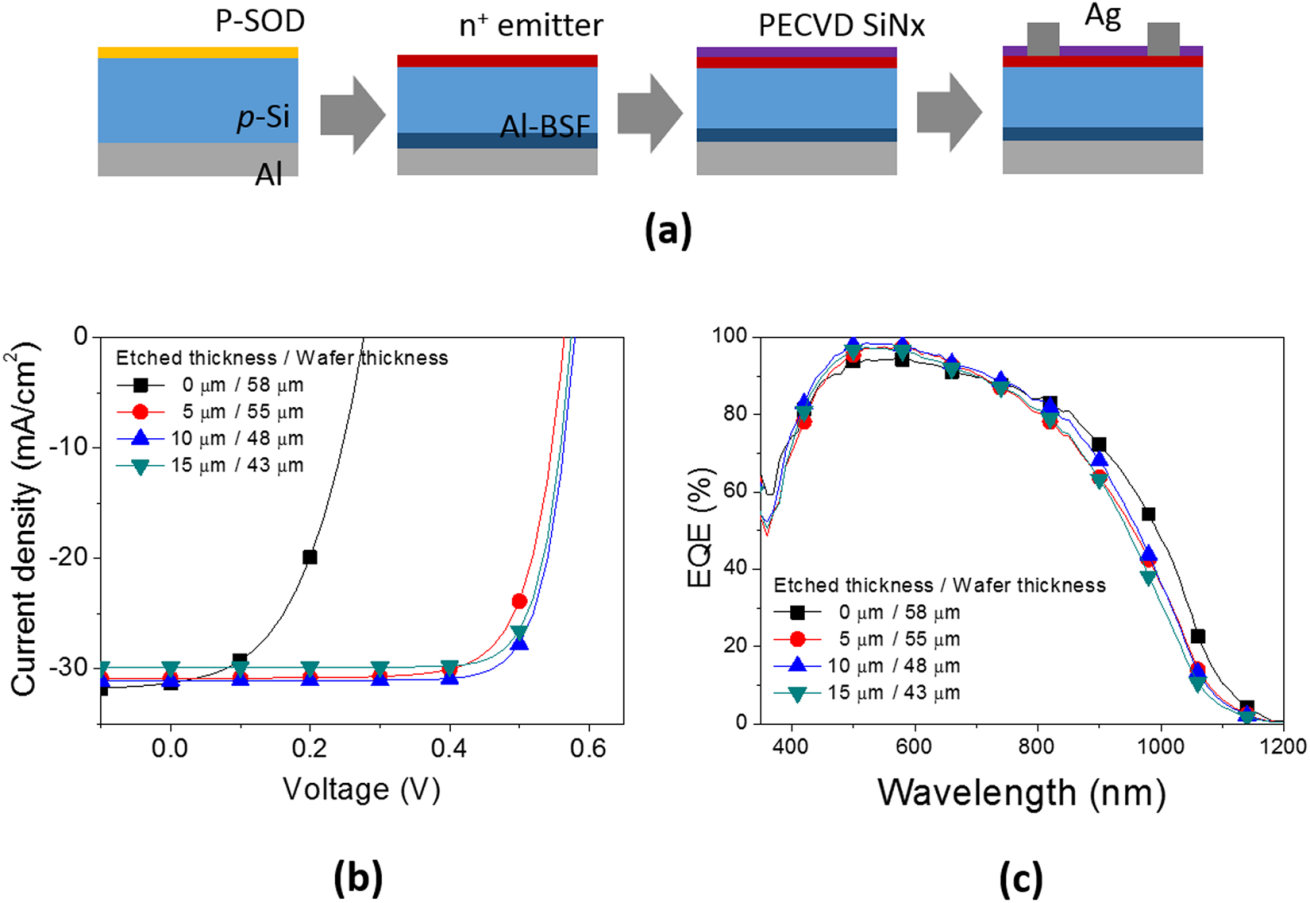
(**a**) Fabrication flow of solar cells based on kerfless planar-wafers without texturing. A standard device architecture with Al back surface field (Al-BSF) is adopted. (**b**) Current-voltage characteristics and (**c**) EQE spectra of the solar cells based on the kerfless wafers which were thinned by chemical etching for implantation damage removal.

The as-cleaved wafers after proton implantation were annealed in N_2_ at 900 °C for 10 min and subsequently etched by varying a removed thickness for minority carrier lifetime recovery. We produced solar cells based on the exfoliated-thin wafers with the cleaved surface as a front side following the process flow shown in Fig. 4(a), and a detailed fabrication process is described in experimental section. No texturing was introduced, and only a SiN_x_ single layer of a 70 nm thickness was deposited for antireflection. Current-voltage characteristic curves and EQE spectra of the solar cells are presented in Fig. 4(b,c).

The solar cell based on the as-cleaved wafer without a damage removal etch exhibits a very low V_oc_ of 0.277 V, which is attributed to increased recombination at the emitter region. The performance parameters of the solar cells are summarized in Table 1. As the damage region at the cleaved side of the wafers is removed by chemical etch, V_oc_ increases dramatically up to 0.564 V only with a 5 μm etch. As the wafer is further etched, V_oc_ and J_sc_ exhibit the maximum of 0.579 V and 31.1 mA/cm^2^ at a 10 μm etch, respectively. The highest efficiency of 14.1% is achieved at a 48 μm thickness. This is comparable to that of the solar cell based on a chemically thinned Si wafer of a 48 μm thickness with a (100) crystal orientation. This result indicates that etching by a thickness of 10 μm at an initial thickness of 58 μm almost completely eliminates implantation damage to the Si wafers.

**Table 1:** Device performance parameters of the solar cells based on the kerfless wafers with etching implantation damages.

Wafer thickness (μm)	V_oc_ (V)	Fill factor	J_sc_ (mA/cm^2^)	Efficiency (%)
58 (as-cleaved)	0.277	0.475	31.3	4.12
53	0.564	0.742	30.9	12.9
48	0.579	0.780	31.1	14.1
43	0.574	0.780	29.9	13.5
reference	0.580	0.780	31.9	14.5

The SiN_x_ layer of a 70 nm thickness is deposited on the planar kerfless wafers for antireflection.

Figure 4(c) shows the EQE values in the short wavelength region below 600 nm improves, supporting the implantation damage is removed by etching. Note that the as-cleave based wafer solar cell shows slightly higher EQE values above the wavelength of 800 nm compared with the etched wafers, which might be caused by the slightly larger thickness of the as-cleaved wafer and also light scattering due to its higher surface roughness.

### Texturing of Si wafers in nano scale for effective light management

Surface texturing of Si wafers for effective light management is crucial to achieve high efficiency^32–34^. The crystal orientation of our kerfless thin wafers is (111); thus, conventional pyramid texturing which works for (100) oriented wafers is not applicable. Furthermore, there is another limitation in texturing scale because the thickness of the kerfless wafer below 50 μm is much thinner than conventional solar grade wafers of 200 μm. In this regard, we developed shallow texturing in sub micrometer scale by combining nanolithography and an isotropic dry etch process.

**Figure 5:**
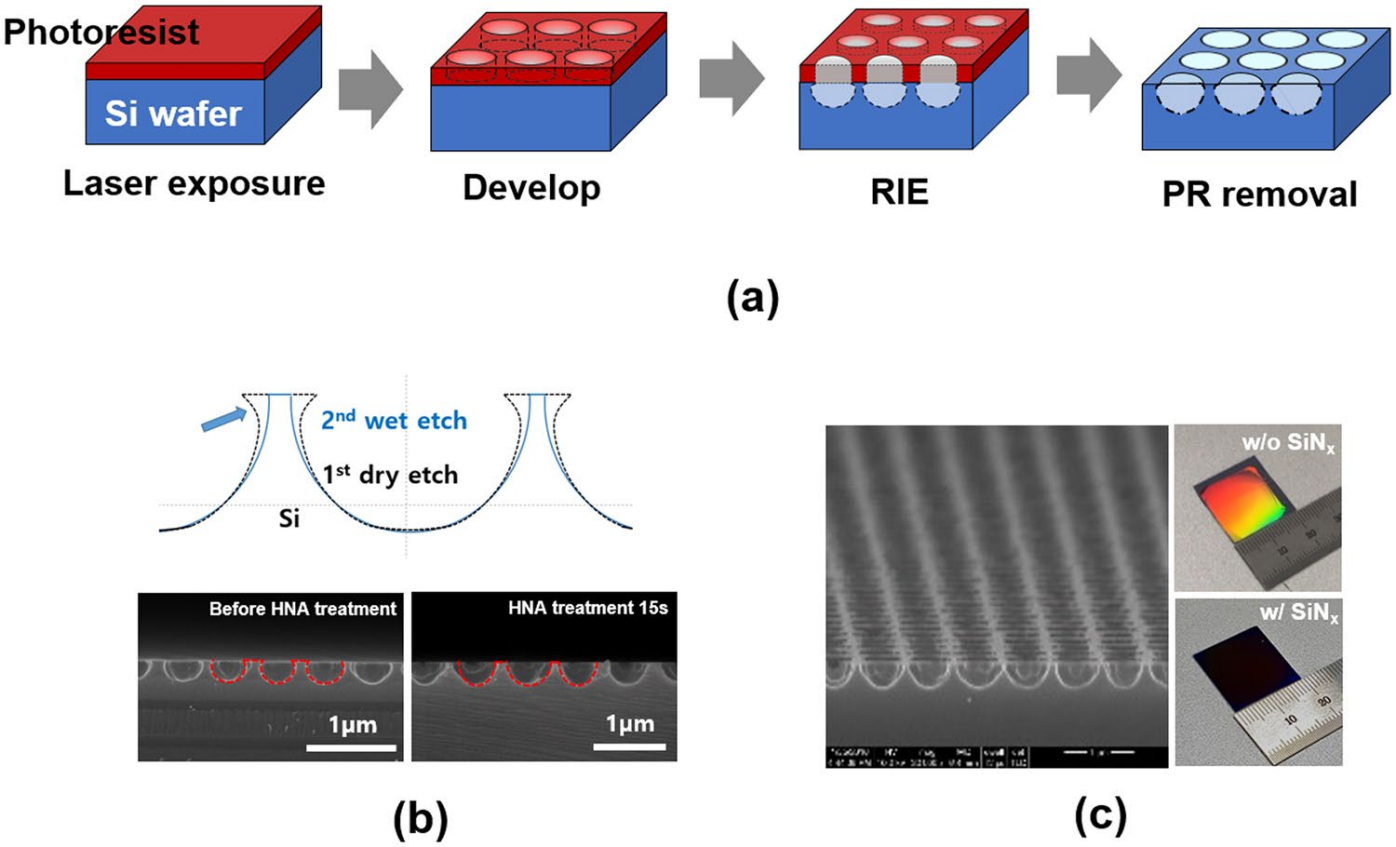
(**a**) Illustrations of a nanohole arrays fabrication process. Photoresist is spun on Si wafers, nanohole arrays are developed by laser interference lithography, Si nanohole arrays are formed by a SF_6_ RIE process, and photoresist is removed. (**b**) Schematic of Si nanoholes in cross-sectional view with a two-step etch process to remove undercut. Cross-sectional SEM images of Si nanohole arrays before and after HNA treatment (bottom left, bottom right). (**c**) Tilted view image of Si nanohole arrays after HNA treatment. Photograph images of Si nanhole arrays without and with SiNx deposition (right).

We applied laser interference lithography to make nanohole arrays in a square lattice with a period of 550 nm. We conducted a SF_6_ RIE process to fabricate periodic Si nanostructures of an inverted nanodome shape of a 300 nm depth. Figure 5(a) illustrates the schematic of the laser interference nano-lithography process. The shape of the nanohole is circular, and the depth is adjusted by a RIE process time. Because of the isotropic RIE etch process, an undercut beneath the photoresist mask is observed, which is detrimental to optical performance and conformal deposition of a passivation layer overcoat. In efforts to remove the undercut, we applied HNA etch for a short time of 15 sec, and the undercut was removed as shown in Fig. 5(b). The good periodicity of the Si nanohole arrays is observed in the tilted view of the SEM image in Fig. 5(c). After the undercut of the nanohole structures is removed, the shape of the nanohole structures in the vertical direction is well matched by the ellipsoid. The Si wafer textured with the nanohole arrays shows strong interference color but after deposition of SiN_x_ of a 70 nm thickness, it becomes nearly black-colored due to a strong antireflection.

**Figure 6:**
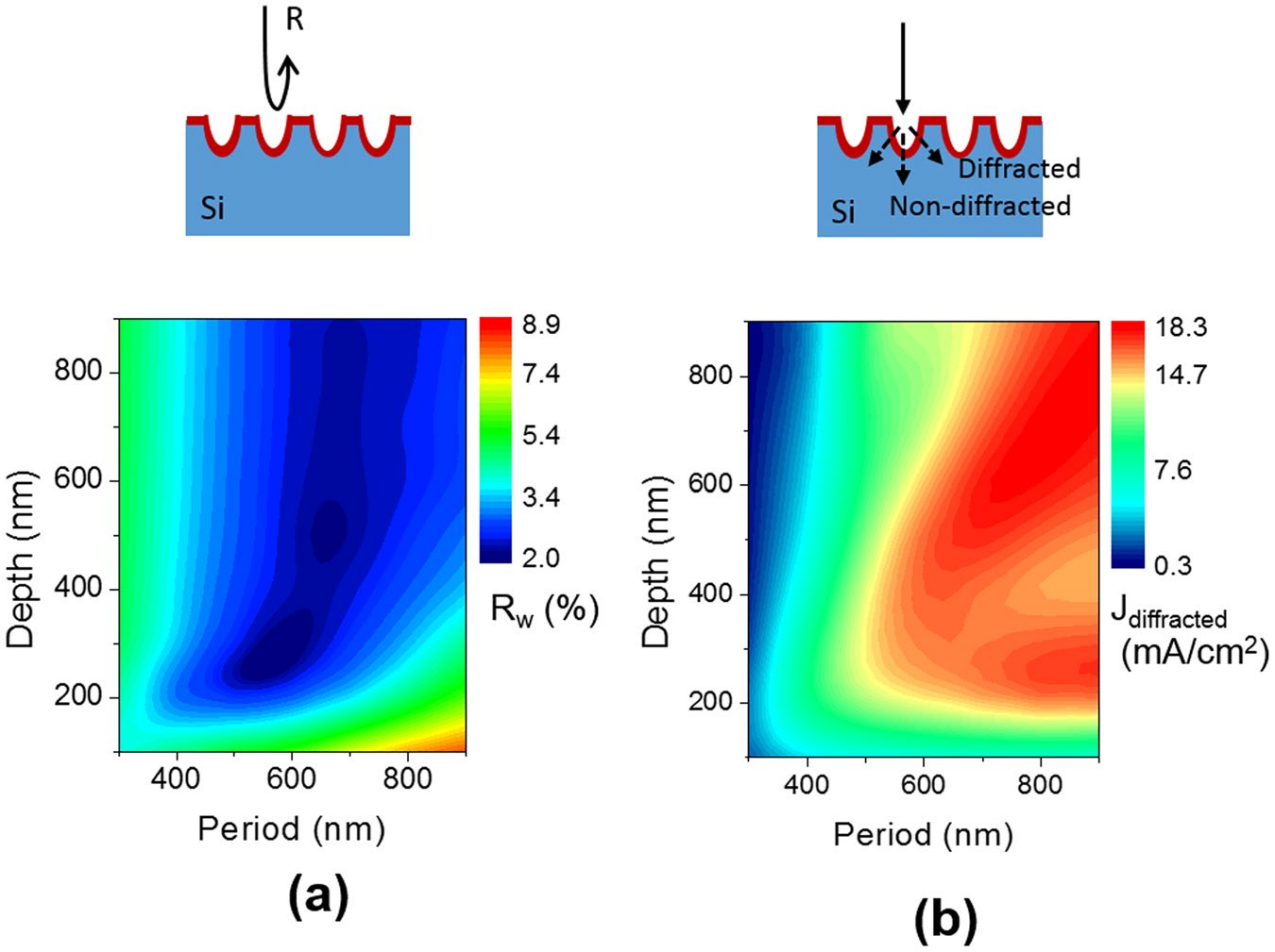
(**a**) RCWA simulated results of weighted total reflectance of the Si nanohole arrays with varying periods and depths. (**b**) Maximum photocurrent of the Si nanohole arrays with varying periods and depths. Only diffracted light in the wavelength from 800 nm to 1200 nm is considered to contribute to photocurrent. The thickness of the Si wafers is assumed to be semi-infinite for both of the calculations.

In order to guide a further optimization of the nanohole arrays in our solar cells, we performed optical simulations by using an RCWA method and the results are illustrated in Fig. 6. The fractional area of the nanoholes at the surface of the Si wafer is fixed at 80% for all the calculations, and an antireflection layer with a refractive index of 1.9 is assumed to be coated over the nanoholes. The periodic Si nanostructures provide multiple effects of graded index and grating diffraction for effective light managements^35^. The average weighted-reflectances were calculated depending on the period and depth of the nanohole arrays and presented in Fig. 6(a). The weighted reflectance (*R*_w_) is calculated by averaging the reflectance (*R*) over a wavelength range from 350 nm to 1200 nm with a weighting of a standard solar irradiation of AM 1.5 G (*I*).

1 $${{\rm{R}}}_{W}=\frac{{\int }_{350nm}^{1200nm}R(\lambda )I(\lambda )d\lambda }{{\int }_{350nm}^{1200nm}I(\lambda )d\lambda }$$

The simulation results show that a 300 nm depth and a 550 nm period of the nanohole arrays provide the lowest reflectance of 2.0%. In addition, diffraction of incident light by periodic nanostructures are beneficial for improving light trapping by enhancing light pathlength of incident light. Because the diffracted light is more effective for light trapping especially in thin Si wafers, only the diffracted light is considered to be collected by excluding the 0^th^ order of transmitted light. Assuming all the photons diffracted into the Si wafer is converted into electricity, a photocurrent (*J*_*diffracted*_) is calculated and shown in Fig. 6(b). Only the wavelengths from 800 nm to 1200 nm, which is a weak absorption range in crystalline Si, are considered for the calculations. In order to achieve a larger *J*_*diffracted*_, a greater period than 500 nm and a deeper depth than 200 nm would be desired.

**Figure 7:**
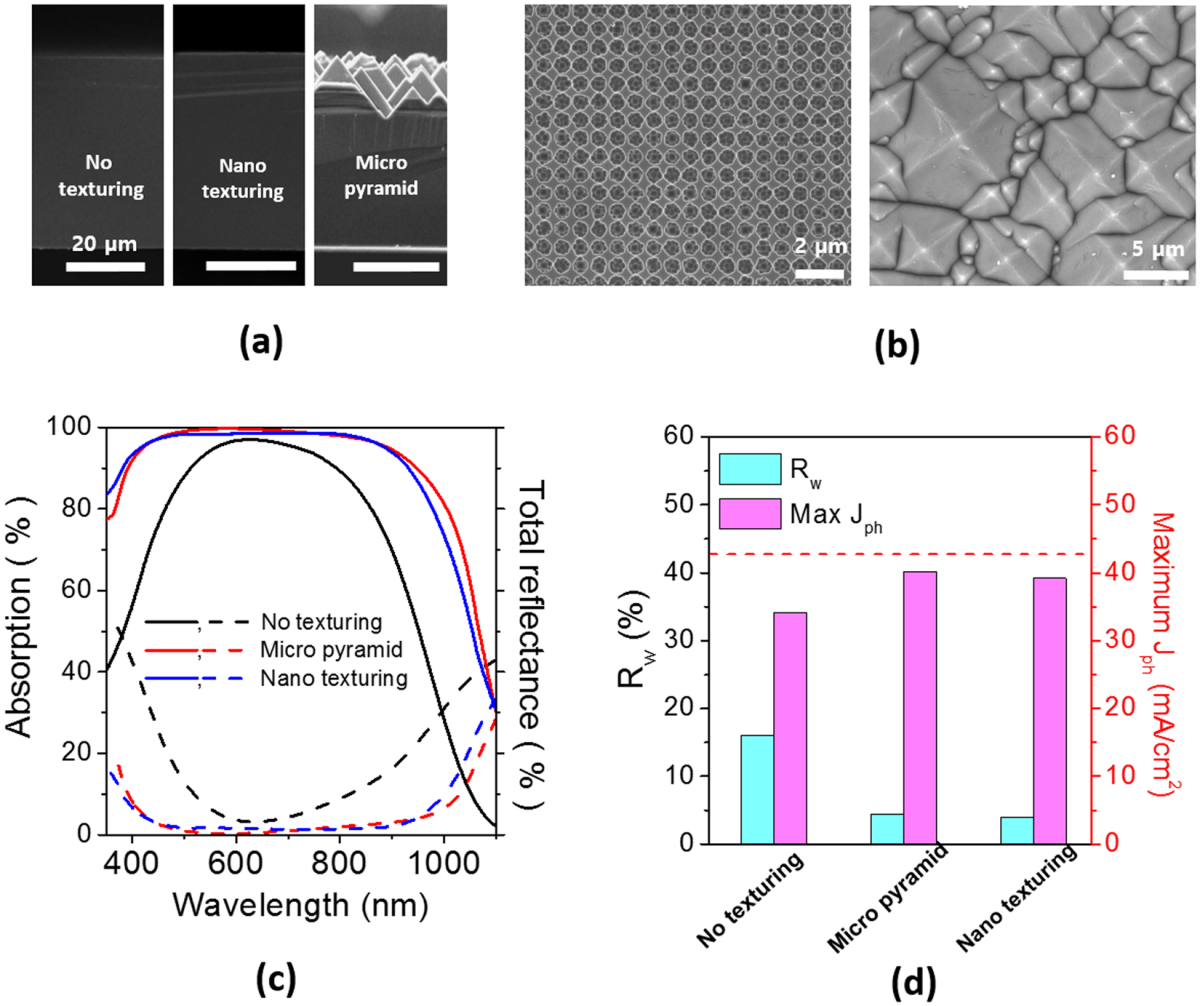
(**a**) Cross-sectional SEM images of the planar, nanohole arrays and micro pyramids. All the scale bars are 20 μm. (**b**) Top-view SEM images of the nanohole arrays and micro pyramids. (**c**) Total absorption and reflectance of thin wafers of a 48 μm thickness with different surface texturing: no texturing, micro pyramids, nanohole arrays. (**d**) Weighted reflectances and maximum photocurrents of the thin Si wafers with different texturing and calculated maximum photocurrents with assuming there is no photon-to-electricity conversion loss.

In order to compare the light trapping efficiency of the nano hole arrays with conventional micro pyramid texturing, we introduced the Si nanohole arrays and micro pyramids on ultrathin wafers of a 48 μm thickness with deposition of SiN_x_. A single layer of SiN_x_ on a planar wafer of the same thickness without texturing was also compared. Figure 7(a) shows the cross-sectional SEM images of three wafers. The micro pyramids ranging 5 μm to 10 μm are clearly observed, whereas the nanohole arrays are not comprehensible in this magnification. The SEM images of nanohole arrays and micro pyramids in a plan view are also shown in Fig. 7(b) for comparison. The total absorptances and reflectances of the wafers with texturing and without texturing. The wafer with micro pyramids exhibits the highest absorptance in a wide spectral range, and the nanohole arrays provide a strong absorptance comparable to micro pyramids. In contrast, the planar wafer with a single layer SiN_x_ shows a high absorptance only in a very narrow spectral range near 600 nm. The wafer with nanohole arrays shows slightly lower *absorptance* in a long wavelength range above 900 nm than that with micro pyramids.

As for a weighted reflectance, the wafer with nanohole arrays exhibits the lowest value of 4.0% in Fig. 7(c). Maximum photocurrents of Fig. 7(d) for three wafers with no texturing, micro pyramids and nanohole arrays were calculated by assuming all the absorbed photons in the wafers contribute to electricity; i.e. internal quantum efficiency is 100%. The ideal limit of the maximum photocurrent with a Lambertian scattering front surface and an ideal rear back reflector is 42.8 mA/cm^2^ as for a wafer of a 48 μm thickness. The ideal photocurrent limit with a Lambertian scattering is calculated by a following absorption enhancement^36^.

2 $${\rm{A}}(\lambda )=\frac{1-\exp (-4\alpha (\lambda )d)}{1-(1-\frac{1}{{n}^{2}})\exp (-4\alpha (\lambda )d)}$$

where *A*(λ) is enhanced absorptance by a Lambertian scattering, *α*(λ) is an absorption coefficient of Si, and *d* is a Si wafer thickness. The wafer with nano hole arrays absorbs 91% of the ideal limit, which is 39.2 mA/cm^2^ in a photocurrent. The micro pyramids provide slightly higher photocurrent of 40.2 mA/cm^2^, which is attributed to strong light trapping in a long wavelength range above 900 nm.

Although the nanohole arrays of our current design provide a lower absorbance than the micro pyramids, a further optimization of the nanohole arrays by tuning their period and depth would lead to enhanced optical performance. We would like to mention that our nano-texturing approach based on laser interference lithography and subsequent RIE would provide a guide for the design of light trapping structures. However, our approach requires costly laser and high vacuum RIE equipment; thus, cost-effective texturing such as metal catalyst-assisted etching can be more practical alternatives^37^.

### Nanohole arrays for kerfless Si solar cells

In order to improve the efficiencies of the solar cells, we modified the device architecture of a standard structure by introducing nano textures at a front side (cell B) and an optical spacer combined with a Ag reflector (cell C) at a rear side. As for the optical spacer, the transparent conducting oxide layer of IZO (indium-zinc-oxide) was chosen to increase an internal reflectance at the rear side of a Si wafer. All the device architectures, an Al back surface field layer was formed by a co-diffusion process, and a metallic Al layer in cell B and C was removed by etching in a boiling HCl solution.

**Figure 8:**
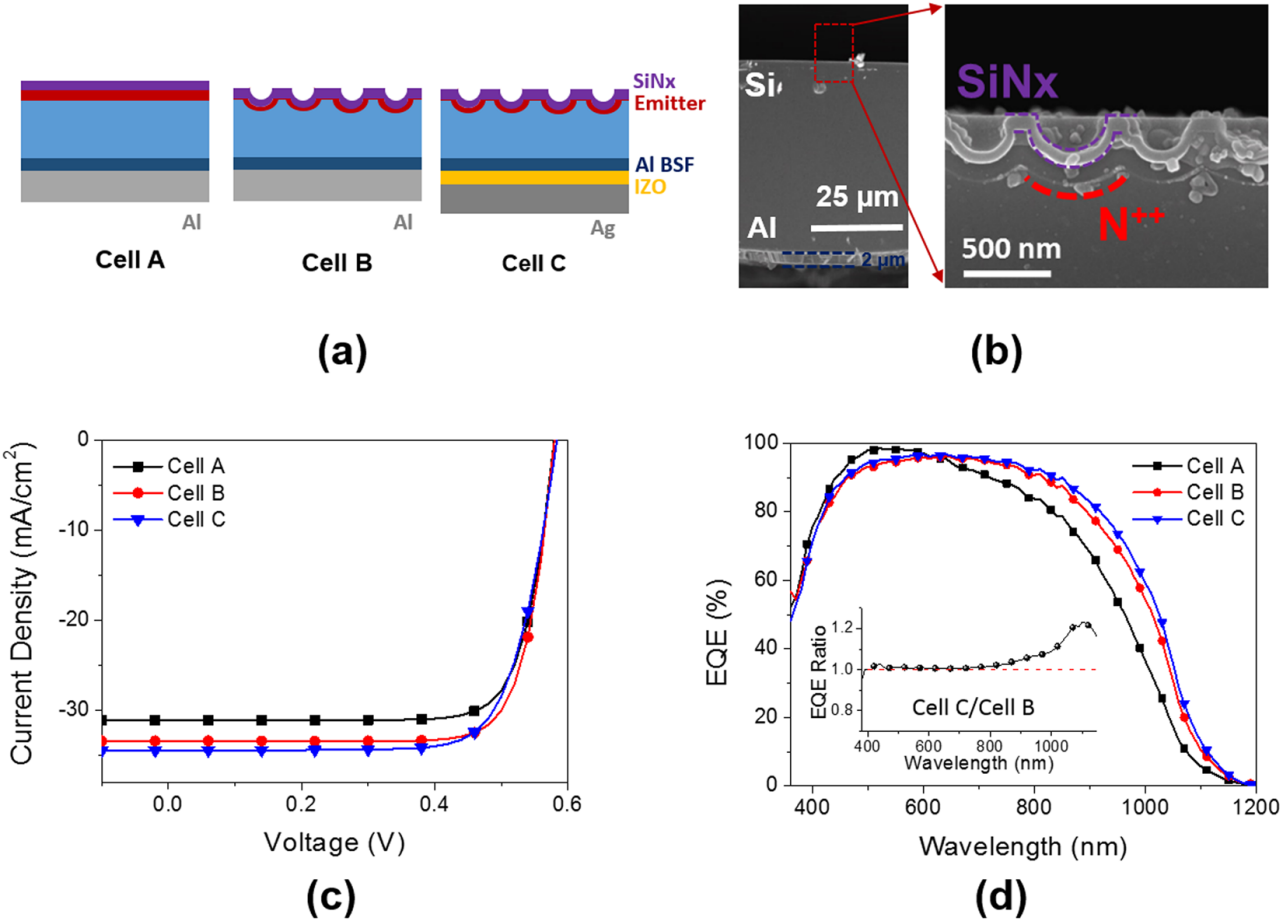
(**a**) Three different device architectures of solar cells based on kerfless wafers. (**b**) Cross-sectional images of the solar cells with the device architecture of cell B. The nanohole arrays on the front side is magnified. A heavily doped region (N^++^) and a SiN_x_ layer are distinctly shown by a selective etching. (**c**) Current-voltage characteristics of the solar cells of three different device architectures under a standard solar irradiation of AM 1.5 G. (**d**) EQE spectra of the solar cells with the different architectures. The inset figure present normalized EQE spectra of cell C to cell B.

Figure 8(a) illustrates schematic figures of three different device architectures. For the nano textures, the nanohole structures developed by laser interference lithography were applied to the front side of the cleaved wafers. Figure 8(b) shows a cross-sectional SEM image of the solar cell based on a kerfless wafer of a 48 μm thickness with a device architecture of cell B. A SiN_x_ layer and a heavily doped emitter region were clearly shown by a selective etch. Current-voltage curves and EQE spectra of three different solar cells under illumination are presented in Fig. 8(c) and (d). As expected, cell B and C exhibit higher photocurrents thanks to enhanced antireflection in broad spectral ranges. As a result, the efficiencies of cell B and C reach 15.2% and 14.9%. Note that cell B has the highest photocurrent of 34.4 mA/cm^2^ among three different cell architectures. The EQE values of cell C is normalized to those of cell B and shown in an inset figure of Fig. 8(d). The EQE values of cell B and C are nearly identical below a wavelength of 800 nm, so the normalized EQE values maintain a unity. However, above the wavelength of 800 nm the normalized EQE values increase consistently up to 1.2. This is attributed to the increased internal reflectance of the rear side by introducing IZO of a 200 nm thickness.

**Figure 9:**
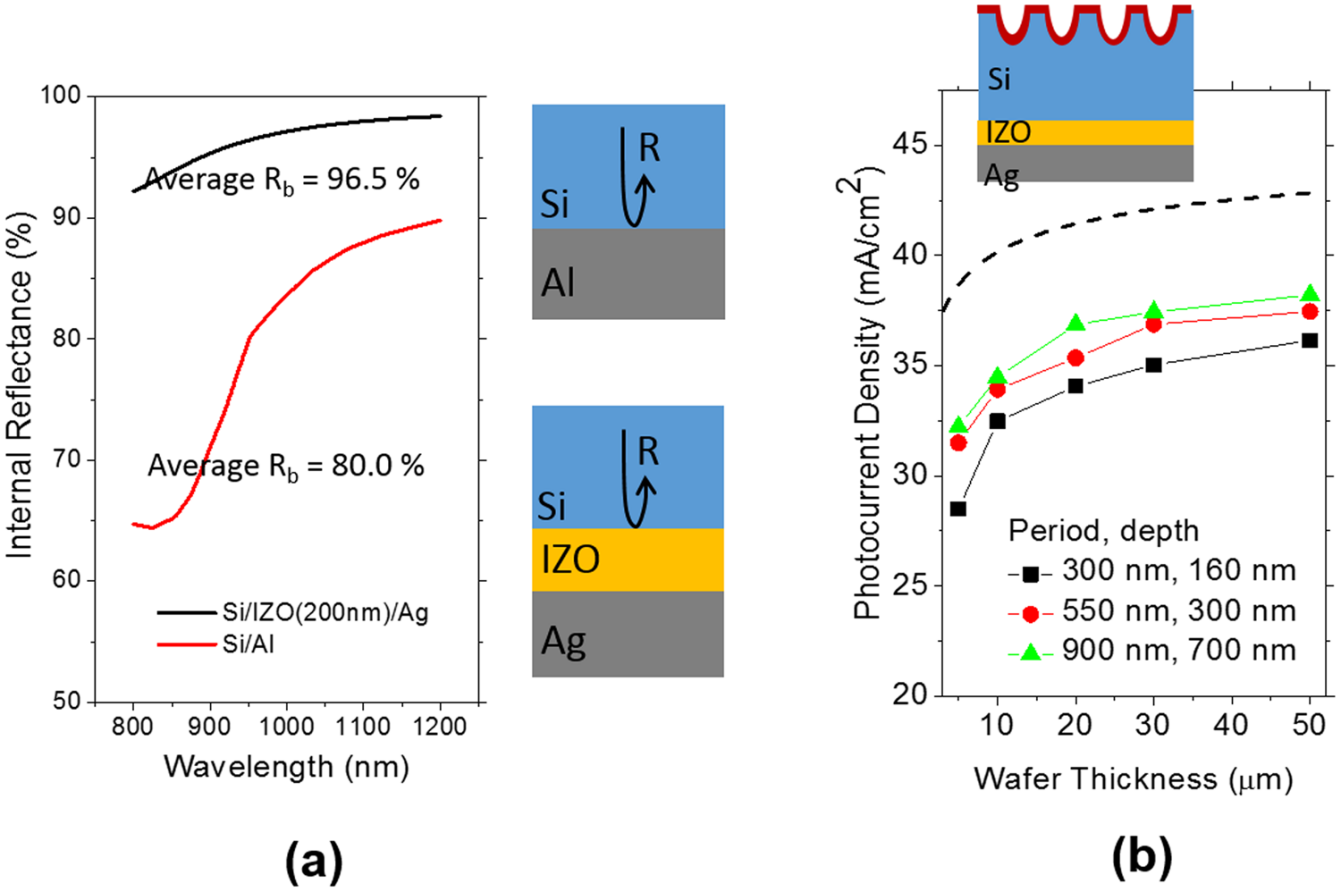
(**a**) Simulated internal reflectance spectra of two different interfaces of Si/Al and Si/IZO/Ag. The incident medium is assumed to have a refractive index of 3.5 which is that of crystalline Si. (**b**) Simulated photocurrents in the Si wafers with the nanohole arrays of different periods and depths as a function of the wafer thickness. The dashed line represents the Lambertian limit of the photocurrents.

The internal reflectances at the interface of Si/Al and Si/IZO/Ag calculated by a thin film optical simulation are 80.0% and 96.5% on average respectively in the wavelength range between 800 nm and 1200 nm as shown in Fig. 9(a). As a thickness of the Si wafers decreases, more photons penetrate into the rear side; thus, higher internal reflectance at the rear side is beneficial for thinner Si wafers. Although cell C provides higher photocurrent than cell B, a FF value of cell C is lower and as a result, its efficiency is slightly reduced compared with cell B. This effect is caused by an increased series resistance, which is considered to result from introduction of a Si/IZO interface. If the contact resistance between Si and IZO can be reduced, then a higher efficiency cab be expected.

Table 2 shows the performance parameters of three different device architectures. We also calculated the photocurrent as a function of a Si wafer thickness with varying a period of the nanohole arrays and presented in Fig. 9(b). The rear reflector is IZO of a 200 nm thickness and Ag as shown in a schematic. The depth of the nanohole arrays was adjusted to scale up with the period. As the wafer thickness becomes thinner, a greater period is more beneficial for light trapping. The nanohole arrays with a 900 nm period provide the highest photocurrent in a given thickness range from 5 μm to 50 μm. The photocurrent of the nanohole arrays with a 900 nm period can reach nearly 89% of Lambertian limit which is the ideal limit denoted by a black dashed line.

**Table 2:** Device performance parameters of the solar cells based on the kerfless wafers of a 48 µm thickness with complete removal of implantation damages.

	V_oc_ (V)	Fill factor	J_sc_ (mA/cm^2^)	Efficiency (%)	R_s_ (Ωcm^2^)
Cell A	0.579	0.780	31.1	14.1	0.60
Cell B	0.580	0.784	33.4	15.2	0.77
Cell C	0.580	0.742	34.4	14.9	1.63

In Summary, we successfully fabricated kerfless thin Si wafers below a thickness of 58 μm with a MeV proton implanter. MCLTs of as-exfoliated kerfless wafers were fully recovered by annealing at high temperature and damage removal by etching of a 5 μm effective thickness per side. The EOR defects induced by proton implantation in exfoliated and donor wafers were confirmed by SEM and TEM observations and exhibited a good agreement with the MCLT measurement results. The position of the ion cut was not in the center of the implantation damage zone and as a result, the EOR defect zone thickness of the exfoliated wafer was much shallower than that of the donor wafer. The Si solar cells of a standard architecture based on the exfoliated wafers were fabricated with etching the implantation damages. The Si solar cell on the exfoliated wafers with a damage removal by a 10 μm etching showed the highest efficiency of 14.1%, which is comparable to the reference cell based on a chemically thinned wafer of a 48 μm thickness, supporting implantation damages were nearly removed. In order to further boost a power conversion efficiency, light trapping structures of Si nanohole arrays produced by laser interference lithography and a dry etch process of SF_6_ RIE were introduced on the kerfless wafers, and as a result, the efficiency was increased up to 15.2%. In efforts to enhance a photocurrent, the rear side was modified to have an optical spacer of transparent conducting oxide and Ag reflector, resulting in a further photocurrent increase.

## Methods

After cleaning the Si donor wafers (CZ, 2 Ωcm, 1 cm × 1 cm) of (111) crystal orientation and a 300 μm thickness by standard RCA1 and RCA2 methods, the Si wafers were implanted with protons of 2.3 MeV and at a dose of 1 × 10^17^/cm^2^. We subsequently annealed the donor wafers after ion implantation at 500 °C for 30 min to have the ultrathin wafers below a thickness of 58 µm exfoliated. Implantation-induced damages to the exfoliated and donor wafers were observed using cross-sectional transmission electron microscopy (X-TEM) prepared by focused ion beam (FIB) machining and scanning electron microscope (SEM). We annealed the kerfless wafers at 900 °C in a nitrogen (N_2_) atmosphere for 10 min to recover point defects. We etched the surface of the donor and kerfless wafers in an acid solution to remove the secondary defects which are not removed by thermal treatment. We introduced a damage removal etching (DRE) process to remove such defects. The DRE solution is a commonly used isotropic wet etchant of a mixture with a volume ratio of HF:HNO_3_:CH_3_COOH (1:75:25)^38–40^. An effective minority carrier lifetime (MCLT) is one of the parameters to represent the wafer quality. We measured the effective MCLT by using µ-PCD (MDP Spot, Freibrug instrument). During MCLT measurements, all the wafers were passivated by iodine and ethyl alcohol solution. We measured the effective MCLT with varying an etched thickness for the cleaved and donor wafers.

The cell fabrication process for a standard device architecture with an Al back surface field is as follows. The first nine step are (1) kerfless wafering by using a PIE technique and cleaning of cleaved wafers by standard clean 1 (RCA1) and standard clean 2 (RCA2), (2) annealing of kerfless wafers in N_2_ at 900 °C for 10 min; 3) damage removal etching in a HNA acid solution, (3) Al deposition at rear side for formation Al back surface field (BSF) by e-beam evaporation, (4) deposition of phosphorus spin-on-dopants (P-SOD) (Filmtronics SOD P507) on the cleaved wafers by spin coating, (5) co-diffusion process at 900 °C for 1 min by rapid thermal process (RTP) and removal of phosphorous silicate glass (PSG) in HF, (6) deposition of Ag front electrodes of a 2 µm thickness with the insertion of Ti buffer layer as adhesion layer by e-beam evaporation, (7) PECVD SiN_x_ 70 nm deposition at 400 °C, (8) edge isolation, and (9) forming gas annealing for 40 min in the tube furnace. In order to enhance an internal reflectance at the rear side, we replaced an Al reflector with transparent conducting oxide and Ag. A metallic aluminum was completely etched in a boiling HCl solution with a remaining Al back surface field layer. As a transparent conducting oxide layer, an indium zinc oxide (IZO) thin film of a 200 μm thickness was deposited by radio frequency sputtering at room temperature.

We introduced periodic nanostructures of square lattice nanohole arrays on the front surface of the thin kerfless Si wafers for effective light trapping. Periodic nanohole arrays were fabricated through laser interference lithography followed by a reactive ion etch process. The period, depth and filling factor of the nanostructures are crucial parameters for effective light trapping in the ultrathin Si wafers. Via the optical simulation method of a rigorous coupled wave analysis (RCWA), we found the optimal design parameters of the inverted nanodome structures for the ultrathin wafers. For the nanohole arrays texturing process, positive photoresist (Dongjin i-7000) was spin coated. Subsequently, two-dimensional (2-D) periodic photoresist nanomask arrays of a square lattice was obtained using laser interference lithography. The square lattice arrays were fabricated by two step exposures of laser. After the first exposure of laser, a photoresist-coated Si wafer is rotated by 90°. The laser source for exposure was a He-Cd laser with a wavelength of 442 nm, manufactured by Kimmon Electric Company. The period of the square lattice arrays can be tuned by adjusting an incidence angle of laser to a substrate coated with photoresist. The process was optimized to prepare a photoresist nanomask with period of about 550 nm. The sample was then etched using a SF_6_ plasma reactive ion etching (RIE) process for 15 seconds to obtain an isotropic shape. The front surface total reflectance and transmittance of ultrathin wafers with the nanohole arrays was measured by a UV-Vis spectrophotometer (Perkin Elmer Lambda 35) with an integrating sphere in the wavelength range of 350 nm −1100 nm. The photovoltaic conversion efficiency and external quantum efficiency of these solar cells were measured by Solar simulator at a light intensity of 100 mW/cm^2^ of a standard AM 1.5 G irradiation and varying the wavelength of the incident monochromatic light from 350 nm to1200 nm respectively.

## Electronic supplementary material

Supplementary Information
