# Supplementary material for: Enhanced efficiency of crystalline Si solar cells based on kerfless-thin wafers with nanohole arrays
Source: Sci Rep. 2018 Feb 22;8:3504. doi: 10.1038/s41598-018-21381-2 (PMC5823930; doi:10.1038/s41598-018-21381-2)
Supplement: Supplementary file 1 — Supplementary Information [file 41598_2018_21381_MOESM1_ESM.doc]

**Supplementary information**

Enhanced efficiency of crystalline Si solar cells based on kerfless-thin wafers with nanohole arrays

Hyeon-Seung Lee^1,2^, Jaekwon Suk^3^, Hyeyeon Kim^3^, Joonkon Kim^3^, Jonghan Song^3^, Doo Seok Jeong^1^, Jong-Keuk Park^1^, Won Mok Kim^1^, Doh-Kwon Lee^4^, Kyoung Jin Choi^5^, Byeong-Kwon Ju^2^, Taek Sung Lee^1^, Inho Kim^1^

*^1^Center for Electronic Materials, Korea Institute of Science and Technology, Seongbuk-gu, Seoul 02792, Republic of Korea*

*^2^ School of Electrical Engineering, Korea University, Seoul 02841, Republic of Korea*

*^3^Advanced Analysis Center, Korea Institute of Science and Technology, Seongbuk-gu, Seoul 02792, Republic of Korea*

*^4^Photo-electronic Hybrids Research Center, Korea Institute of Science and Technology, Seongbuk-gu, Seoul 02792, Republic of Korea*

*^5^School of Materials Science and Engineering, Ulsan National Institute of Science and Technology (UNIST), Ulsan 44919, South Korea*

**Methods**

The first kerfless-thin wafer (1 cm × 1cm) was fabricated with proton implantation at a dose of 1×10^17^/cm^2^ and subsequent heat treatment for exfoliation in the same manner described in the main manuscript. The donor wafers were re-used for the second exfoliation of the kerfless-thin wafers. The acceleration voltage and dose for proton implantation and the heat treatment condition were the same as the first exfoliation process. In the second cycle of the exfoliation, the as-cleaved donor wafers were used without any surface polishing. The thickness and surface roughness of the kerfless-thin wafers were analyzed by SEM and AFM measurements. The overall process for the kerfless wafering of the second cycle is illustrated in Figure S1.


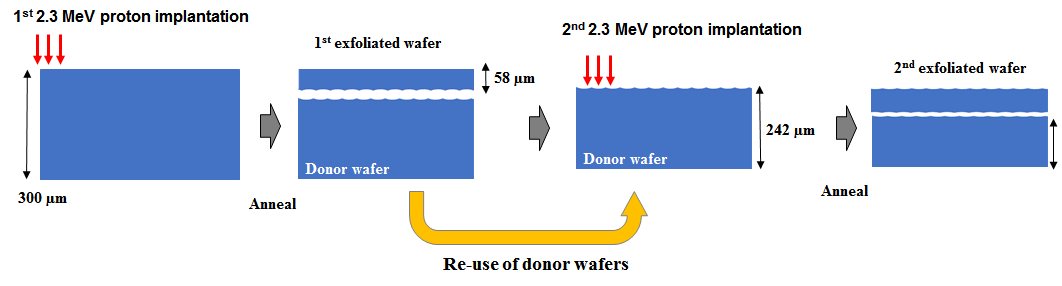


Figure S1. Fabrication process of the kerfless-thin wafers using the reused donor wafers.

**Results**

The second kerfless-thin wafers of a 1cm × 1cm size were successfully exfoliated from the re-used donor wafers. The photographs of the kerfless-thin wafers were taken and shown in Figure S2 (a). The cross-sectional SEM images were also taken to confirm the thickness of the kerfless-thin wafers as shown in Figure S2 (b). The thickness of the second exfoliated wafer was the same as the first kerfless-thin wafer of a 58 μm thickness. The thickness of the donor wafer was reduced to 184 μm after two cycles of implant and exfoliation.


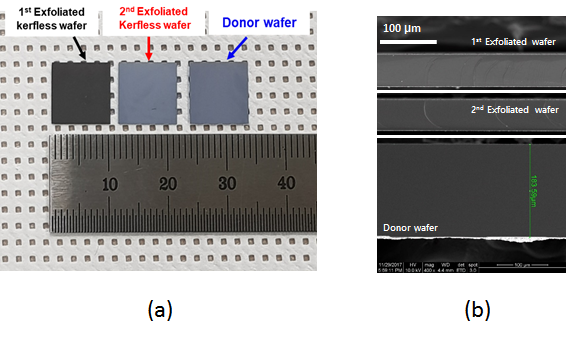


Figure S2. (a) Photographs of the kerfless wafers fabricated with the first and second cycles of implant and exfoliation. The donor wafer is also shown together after the second exfoliation cycle. The rear sides of the kerfless wafers and the front side of the donor wafer are shown. (b) The cross-sectional SEM images of the kerfless-thin and donor wafers.

The AFM images were taken for the second exfoliated-thin wafer in order to investigate whether there would be an increase in the surface roughness of the exfoliated wafers with increasing wafering cycles. The donor wafer has initially a polished surface which is flat in an atomic scale. After the first exfoliation, the front surface of the donor wafer has an rms roughness of ~70 nm. However, the rms roughness of the second exfoliated-thin wafer at the front side slightly increases up to 112 nm as shown in Table S1. The rear side of the second exfoliated-thin wafer has a similar rms roughess as the firs exfoliated one, and the front side of the donor wafer after the second exfoliation does also. The reason the front surface roughness of the second kerfless wafer increased would relate to thermal treatment for exfoliation. It seems structural defects at the cleaved side evolve during the exfoliation process. However, the increased rms roughness at the front side does not appear to influence on the rear side rms roughness during exfoliation. How many times the donor wafer can be reused? The maximum number of the donor wafer recycle is one of the important issues for exfoliation wafering. This would be another good research topic for further study.


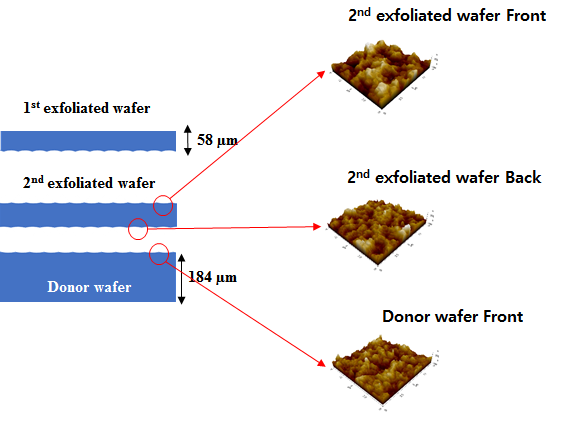


Figure S3. AFM surface height images of the kerfless wafers and donor wafer.

Table S1. Average rms roughness of the kerfless-thin wafers and donor wafer.

|  | rms roughness (nm) |
| --- | --- |
| The front side of the second exfoliated-thin wafer | 111.6 |
| The rear side of the second exfoliated-thin wafer | 79.5 |
| The front side of the donor wafer | 74.0 |
